# Odorant-Binding Proteins of the Malaria Mosquito *Anopheles funestus sensu stricto*

**DOI:** 10.1371/journal.pone.0015403

**Published:** 2010-10-22

**Authors:** Wei Xu, Anthony J. Cornel, Walter S. Leal

**Affiliations:** Department of Entomology, University of California Davis, Davis, California, United States of America; Griffith University, Australia

## Abstract

**Background:**

The mosquito *Anopheles funestus* is one of the major malaria vector species in sub-Saharan Africa. Olfaction is essential in guiding mosquito behaviors. Odorant-binding proteins (OBPs) are highly expressed in insect olfactory tissues and involved in the first step of odorant reception. An improved understanding of the function of malaria mosquito OBPs may contribute to identifying new attractants/repellents and assist in the development of more efficient and environmentally friendly mosquito controlling strategies.

**Methodology:**

In this study, a large screening of over 50 ecologically significant odorant compounds led to the identification of 12 ligands that elicit significant electroantennographic (EAG) responses from *An*. *funestus* female antennae. To compare the absolute efficiency/potency of these chemicals, corrections were made for differences in volatility by determining the exact amount in a stimulus puff. Fourteen AfunOBP genes were cloned and their expression patterns were analyzed. AfunOBP1, 3, 7, 20 and 66 showed olfactory tissue specificity by reverse transcriptase PCR (RT-PCR). Quantitative real-time PCR (qRT-PCR) analysis showed that among olfactory-specific OBPs, AfunOBP1 and 3 are the most enriched OBPs in female antennae. Binding assay experiments showed that at pH 7, AfunOBP1 significantly binds to 2-undecanone, nonyl acetate, octyl acetate and 1-octen-3-ol but AfunOBP3, which shares 68% identify with AfunOBP1 at amino acid level, showed nearly no binding activity to the selected 12 EAG-active odorant compounds.

**Conclusion:**

This work presents for the first time a study on the odorants and OBPs of the malaria vector mosquito *An. funestus*, which may provide insight into the *An. funestus* olfactory research, assist in a comparative study between major malaria mosquitoes *An. gambiae* and *An. funestus* olfactory system, and help developing new mosquito control strategies to reduce malaria transmission.

## Introduction

Malaria, the most serious mosquito-borne infectious disease, is widely distributed in the tropical and subtropical regions of the world. According to the last annual world-wide malaria morbidity and mortality survey in 2006, there were about 247 million cases among 3.3 billion people at risk, causing almost a million deaths, mostly in children under 5 years [1]. The principal malaria vector species in sub-Saharan Africa are *Anopheles gambiae sensu stricto* and *Anopheles funestus sensu stricto* [2], [3], which belong to the same subgenus *Cellia* and diverged from a common ancestor approximately 5 million years ago [4]. Indeed, despite differences in morphology, breeding site preferences, mating behavior and relative seasonal abundance, both species coexist geographically in many parts of sub-Saharan Africa and both are highly anthropophilic and endophilic [2], [3].

Despite extensive research conducted on the behavior, ecology and genomics of *An. gambiae*, much less is known about *An. funestus* since until very recently colonies of this latter species were unavailable. Vector control strategies must also impact and reduce *An. funestus s.s* populations to reduce overall malaria incidence. In 2002, the genome project of *An. gambiae s.s* was completed thus providing an invaluable resource to conduct comparative genetic and phenotype association studies among several *Anopheles* malaria vectors [5]. Many of these association studies include mechanisms of phenotypes that affect vector capacities of these malaria mosquito species, which will create new avenues for vector control strategies. Novel control strategies are needed as resistance to currently used pyrethroid insecticides have been recorded in both *An. gambiae* [6] and *An. funestus* [7].

Olfaction is essential in guiding insect behaviors such as foraging, host-seeking, and oviposition [8]. In female mosquitoes a population of hair-like sensilla distributed over the surface of the antennae and maxillary palps act as a nose to detect chemical signals. The major proteins involved in the selectivity and sensitivity of the insect olfactory system are odorant-binding proteins (OBPs) [9], [10] and odorant receptors (ORs) [11]. OBPs are involved in the first step of odorant reception where they bind, solubilize and deliver odorant molecules to ORs [10]. ORs are heterodimers comprised of highly variable odorant-binding subunits associated with one conserved co-receptor (OR83b) and localized on the dendrite membrane in the olfactory sensilla [12], [13]. They detect odorant compounds and transduce olfactory signals to the brain to mediate insect behaviors.

The first insect OBP was discovered at the beginning of 1980s in the giant moth *Antheraea polyphemus* [9] while the first mosquito OBP (CquiOBP1) was isolated from antennae of female *Culex quinquefasciatus* in 2002 [14]. The release of the genome sequences of several mosquito species such as *An. gambiae*, *Aedes aegypti* and *Cx. quinquefasciatus* has allowed the identification of large multigenic families of OBPs. To date, 33 classic OBPs have been identified in *An. gambiae* [15], [16], [17]; 34 classic OBPs were identified in *Ae. aegypti* [18], and 55 classic OBPs were identified in *Cx. quinquefasciatus* [19].

The literature documents various roles for insect OBPs. LUSH is a soluble OBP of the fruit fly *Drosophila melanogaster.* Deletion of LUSH gene suppresses *D. melanogaster* electrophysiological and behavioral response to the male pheromone 11-cis-vaccenyl acetate (cVA) [20]. Octanoic and hexanoic acids, two odorant compounds originate from the *Morinda citrifolia*, act as oviposition attractants for *D. sechiella* but as repellents for *D. melanogaster* [21]. Deleting OBP57d and OBP57e genes in *D. melanogaster* eliminates the avoidance behavior, while reinserting the orthologous genes of *D. sechiella* into *D. melanogaster* results in attraction to these two fatty acids [21]. *Bombyx mori* pheromone binding protein (BmorPBP1) and pheromone receptor BmorOR1 were expressed in an "empty neuron" system of a *Drosophila* mutant and the response to the *B. mori* pheromone bombykol was analyzed [22]. Flies carrying both BmorPBP1 and BmorOR1 showed significantly higher electrophysiological responses than flies carrying BmorOR1 only [22]. Recently, two RNAi-mediated OBP genes silencing coupled with electrophysiological analyses have demonstrated the importance of OBPs in odorant recognition in two mosquito species [23], [24]. By knocking down CquiOBP1 in *Cx. quinquefasciatus*, mosquitoes showed reduced antennal response to several oviposition attractants compared to controls [23]. Likewise, after injecting AgamOBP1 double-strand RNA into *An. gambiae*, mosquito response to indole were impaired [24]. All these studies showed that OBPs are critical for the selectivity and sensitivity of insect olfactory system. Therefore, the study on OBPs of malaria vector *An. funestus* might help us understanding the molecular basis of olfaction in this species and developing environmentally friendly strategies for mosquito control.

In this study, we have identified for the first time 12 odorants that elicit significant EAG responses and, therefore, may be potential attractants or repellents for *An. funestus* mosquito. To compare EAG activities of these compounds on a molar basis, corrections were made for differences in volatility by determining the exact amount in a stimulus puff. In addition, 14 AfunOBP genes were identified by homology cloning based on the published classic OBP sequences and their expression patterns were determined. Two most female abundant olfactory tissue specific OBPs, AfunOBP1 and AfunOBP3, were expressed, purified, characterized, and their affinity towards EAG active compounds was examined by using binding assay experiments. AfunOBP1 showed binding activity to 2-undecanone, nonyl acetate, octyl acetate and 1-octen-3-ol. AfunOBP3, an OBP sharing 68% identity with AfunOBP1 at amino acid level, showed nearly no binding activity to the selected 12 EAG active odorant compounds. This work presents for the first time a study on the odorants and OBPs of the malaria vector mosquito *An. funestus*, which may provide insight into *An. funestus* olfactory research, assist in a comparative study between the olfactory systems of *An. gambiae* and *An. funestus*, and help developing new mosquito control strategies to reduce malaria transmission.

## Results and Discussion

### EAG study

**Figure 1:**
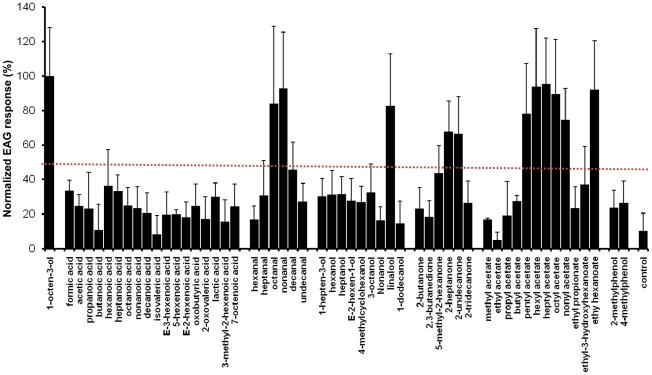
Responses of female *An. funesuts* antennae to various human and plant derived chemicals. Source dose, 100 µg, n = 3–20; mean ± std, age: 1–6 day-old. EAG response of 1-octen-3-ol was used as a standard (100%) to normalize EAG responses elicited by other chemicals. Hexane was used as a control. Octanal, nonanal, linalool, 2-undecanone, 2-heptanone, pentyl acetate, hexyl acetate, heptyl acetate, octyl acetate, nonyl acetate and ethyl hexanoate elicited more than 50% of the 1-octen-3-ol response.

**Figure 2:**
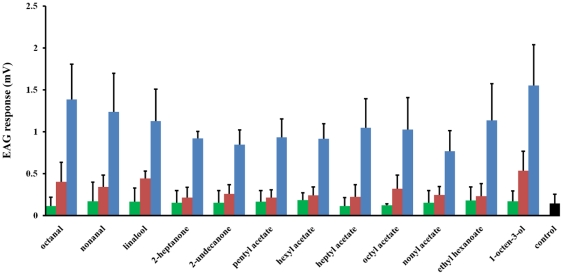
Dose-dependent EAG responses recorded from female *An. funestus* antennae. Source doses of selected ligands: 1 µg (green), 10 µg (red) and 100 µg (blue), n = 5; mean ± std. Hexane was used as a control.

Our initial screening identified 12 odorants that elicited significant antennal responses from female *An. funestus* at 100 µg source dose (Fig. 1). By selecting 1-octen-3-ol as a standard, we identified octanal, nonanal, linalool, 2-undecanone, 2-heptanone, pentyl acetate, hexyl acetate, heptyl acetate, octyl acetate, nonyl acetate and ethyl hexanoate as the best ligands, i.e., compounds which elicited more than 50% of 1-octen-3-ol response. These 11 compounds, along with the standard 1-octen-3-ol, were further evaluated for their dose-dependent EAG response at 1, 10 and 100 µg source dose (Fig. 2). Chemicals screened in this study included compounds known to elicit significant antennal responses from *An. gambiae*, specifically formic acid, acetic acid, propanoic acid, lactic acid, p-cresol, 1-octen-3-ol, 7-octenoic acid, 3-methyl-2-hexenoic acid [25], [26]. However, when tested on *An. funestus* these compounds, except 1-octen-3-ol, did not elicit significant EAG responses. This discrepancy between *Anopheles* species may be attributed to ecological differences between these two malarial vectors. Indeed, *An. funestus* differs markedly from *An. gambiae* in breeding site preferences, mating behavior and relative seasonal abundance [4].

1-Octen-3-ol was originally identified from the analysis of cattle odor [27] and later from human sweat [25]. It has been shown to serve as a powerful attractant for certain species of testse flies in the field [27]. Subsequent studies on mosquitoes have revealed that 1-octen-3-ol affects host-seeking behavior in mosquitoes [28], [29], [30]. Traps baited with 1-octen-3-ol resulted in moderate catch increase of a few mosquito species, but in combination with CO_2_, 1-octen-3-ol serves as an efficient attractant [31]. Octanal and nonanal were both identified from human [32], [33] and the latter has recently been shown as a major compound from both human and bird, which are alternate hosts of *Cx. quinquefasciatus* [33]. This compound elicited significant electrophysiology responses from female *Cx. quinquefasciatus* and nonanal baited traps added with CO_2_ led to significantly higher catches of *Culex* mosquitoes [33]. 1-Octen-3-ol, octanal and nonanal were all identified from human, thus these compounds may be associated with the anthropophilic behaviors in *An. funestus*.

Interestingly, many floral and plant compounds viz., pentyl acetate, hexyl acetate, heptyl acetate, octyl acetate, nonyl acetate, ethyl hexanoate and 2-heptanone elicited high EAG activity in female *An. funestus*. These compounds may be involved in mosquito sugar-feeding behaviors. Sugar is the basic source of nutrients which provide energy to mosquitoes [34]. A ketone, 2-undecanone, was originally identified from wild tomato [35] and was later shown to have repellent activity against mosquitoes and ticks [36], [37], [38]. Linalool is a natural compound found in many flowers [39] and was also reported for its repellent activity on mosquito [40]. Thus, 2-undecanone and linalool that elicit high EAG responses in An. funestus may also be repellents for this mosquito species.

**Figure 3:**
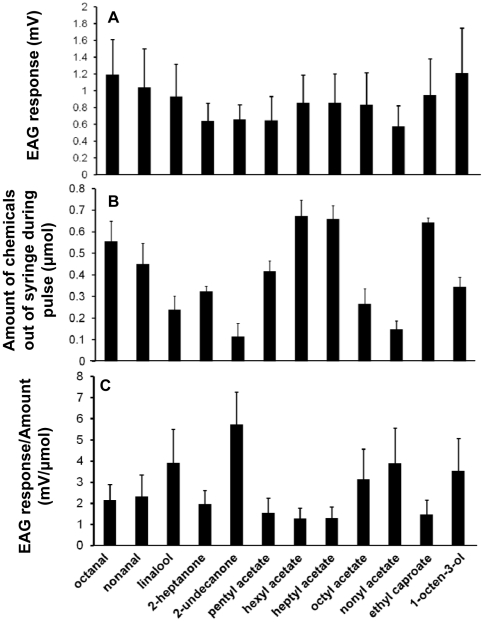
Relative EAG responses. (**A**) EAG responses (mV) of 12 selected chemicals at 100 µg source dose to the female *An. fuenstus* antennae (n = 5; mean ± std); (**B**) amount of the odorants released from the EAG syringe during stimulus (n = 10; mean ± std); (**C**) EAG response per micro molar of odorant (mV/µmol). The highest value on EAG response/µmol (mV/µmol) was recorded with 2-undecanone.

To overcome the difference in the volatility of test ligands during a stimulus [41], the amounts of chemicals released from the EAG syringe were quantified for the twelve compounds that elicited high EAG responses (Fig. 3A,B). The experiment showed that the amount of 2-undecanone released from the EAG syringe during stimulus was much less than the other chemicals as expected due to its larger molecular weight. To better correlate EAG response and the released amount of these odorants, we calculated the “EAG response/µmol” (Fig. 3C). The highest EAG response/µmol (mV/µmol) value was obtained for 2-undecanone. An arthropod repellent named BioUD, which was registered by US Enviornmental Protection Agency (EPA) *in* 2007, contains 7.75% of 2-undecanone. It was proposed to function as an effective alternative to DEET because of its repellent activity to mosquitoes [37].

### Identification of fourteen putative OBP genes in *Anopheles funestus*

**Figure 4:**
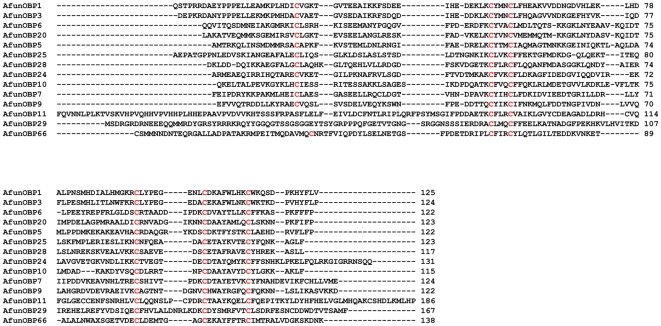
Alignment of amino acid sequences of newly identified OBPs from *An. funestus*. Six conserved cysteine residues are highlighted in red.

We have employed a homology cloning strategy based on previously identified *An. gambiae* OBP genes to isolate putative orthologs in *An. funestus*. We were able to clone fourteen genes, *AfunOBP1, AfunOBP3, AfunOBP5, AfunOBP6, AfunOBP7, AfunOBP9, AfunOBP10, AfunOBP11, AfunOBP20, AfunOBP24, AfunOBP25, AfunOBP28, AfunOBP29* and *AfunOBP66* which display high identity to their respective orthologs from *An. gambiae*. An alignment of mature AfunOBPs amino acid sequences highlights the high overall divergence of this family as only the six cysteine residues are completely conserved between all proteins (Fig. 4). Except for AfunOBP11 and AfunOBP29, the other AfunOBPs share the characteristic features of the classic OBP family, namely, small size, presence of a N-terminal signal peptide sequence as well as a highly conserved pattern of six cysteine residues called the “classic motif” [19] (Fig. 4). AfunOBP11 molecular weight is about 21 kDa (Table 1) and it contains 12 cysteine residues, whereas AfunOBP29 molecular weight is over 19 kDa and no signal peptide was predicted. Additionally, both proteins do not satisfy the classic motif of cysteine spacing [18] and Conserved Domain Database (CDD) prediction also showed that these two protein values for PBP/GOBP family are the lowest (Table 1) in all 14 AfunOBPs. However, their respective orthologous genes in *An. gambiae*, AgamOBP11 and AgamOBP29, were classified as “classic” OBPs in previous studies [17].

**Table 1:** List of 14 cloned *An. funestus* OBPs parameters.

OBP Name	GenBank accession #	Amino Acids	MW	pI	Cysteine Spacing	Signal Peptide %	CDD prediction(E-value)
AfunOBP1	HM436669	144/125	14526	5.53	26/3/37/8/8	99.9	PBP_GOBP (1e_22)
AfunOBP3	HM436670	151/124	14618	5.30	26/3/37/8/8/12	89.1	PBP_GOBP (5e_25)
AfunOBP5	HM436671	151/123	13988	8.76	26/3/41/10/8	98.6	PBP_GOBP (8e_25)
AfunOBP6	HM436672	160/122	13983	6.76	26/3/39/10/8	67.3	PBP_GOBP (4e_16)
AfunOBP7	HM436673	142/124	14287	5.06	13/12/42/8/8/11	97.2	PBP_GOBP (1e_16)
AfunOBP9	HM436674	139/122	14010	4.97	26/3/38/9/8	100	PBP_GOBP (2e_19)
AfunOBP10	HM436675	136/115	13018	8.50	28/3/38/7/8	99.9	PBP_GOBP (5e_11)
AfunOBP11	HM436676	207/186	21228	6.42	26/3/8/10/7/0/9/7/3/8/24	99.9	PBP_GOBP (0.004)
AfunOBP20	HM436677	136/121	13488	7.72	26/3/40/10/8	5.5	PBP_GOBP (6e_17)
AfunOBP24	HM436678	156/131	14959	8.76	27/3/38/7/8	88.5	PBP_GOBP (2e_12)
AfunOBP25	HM436679	144/123	13578	4.66	27/3/38/7/8	99.9	PBP_GOBP (3e_10)
AfunOBP28	HM436680	131/115	12981	5.94	28/3/39/7/8	100	PBP_GOBP (5e_09)
AfunOBP29	HM436681	167	19343	5.85	3/42/13/8/8	0	PBP_GOBP (0.005)
AfunOBP66	HM436682	166/138	15470	4.45	35/29/3/33/8/8/15	99.3	PBP_GOBP (3e_04)

**Figure 5:**
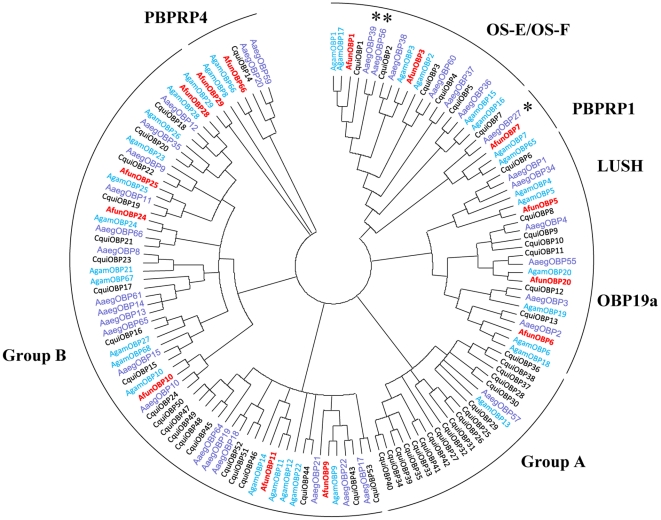
Phylogenic analysis of mosquito OBP amino acid sequences. 33 classic AgamOBPs (blue), 53 classic CquiOBPs (black), 34 classic AaegOBPs (purple), and new cloned 14 AfunOBPs (red) were grouped into OS-E/OS-F, PBPRP1, LUSH, OBP19a, PBPRP4, Group A and Group B.

Each OBP from *An. funestus* belongs to different groups of orthologous proteins previously described (Fig. 5) [19]. Two proteins (AfunOBP1 and AfunOBP3) belong to the OS-E/OS-F group, one (AfunOBP7) to the PBPRP1 group, one (AfunOBP5) to the LUSH group, two (AfunOBP6 and AfunOBP20) to the OBP19a group, and one (AfunOBP66) to the PBPRP4 group. All the other 7 *An. funestus* OBPs are clustered into group B. Each AfunOBP is more closely related to putative orthologous OBPs in *An. gambiae* than to putative orthologous OBPs from other mosquito species (Table 2), as expected by *An. funestus* being more closely related to *An. gambiae* than to other mosquito species. Thirty-three “classic” OBPs have been identified from *An. gambiae* [15], [16], [17], thirty-four and fifty-three “classic” OBPs have been identified from *Ae. aegypti* [18] and *Cx. quinquefasciatus* [19], respectively. It is likely that more OBPs are present in *An. funestus* as the genome project of this species is not yet available and may provide more information about the complete repertoire of OBPs.

**Table 2:** List of OBPs from *An. funestus* OBPs and their orthologues from other mosquito species.

OBP	Phylogenetic group	*An. gambiae* homolog	Protein identity	*A. aegypti* homolog	Protein identity	*Cx. quinquefasciatus* homolog	Protein identity
AfunOBP1	OS-E/OS-F	AgamOBP1	93%	AaegOBP1	85%	CquiOBP1	90%
AfunOBP3	OS-E/OS-F	AgamOBP3	95%	AaegOBP38	90%	CquiOBP2	87%
AfunOBP5	LUSH	AgamOBP5	91%	AaegOBP1/34	59%/54%	CquiOBP6	62%
AfunOBP6	OBP19a	AgamOBP6/18	83%/82%	AaegOBP2	65%	CquiOBP13	64%
AfunOBP7	PBPRP1	AgamOBP7	92%	AaegOBP27	65%	CquiOBP7	64%
AfunOBP9	Group B	AgamOBP9	91%	AaegOBP22	70%	CquiOBP43	76%
AfunOBP10	Group B	AgamOBP10	84%	AaegOBP10	64%	CquiOBP24	66%
AfunOBP11	Group B	AgamOBP11	79%	AaegOBP40	28%		
AfunOBP20	OBP19a	AgamOBP20	93%	AaegOBP55	70%	CquiOBP11	74%
AfunOBP24	Group B	AgamOBP24	88%	AaegOBP66	36%	CquiOBP21	
AfunOBP25	Group B	AgamOBP25	87%	AaegOBP11	59%	CquiOBP19	59%
AfunOBP28	Group B	AgamOBP28	88%	AaegOBP12	58%	CquiOBP18	62%
AfunOBP29	Group B	AgamOBP29	68%				
AfunOBP66	PBPRP4	AgamOBP66	79%	AaegOBP59/20	60%/59%	CquiOBP14	56%

### Expression profiles of *An. funestus* OBPs

**Figure 6:**
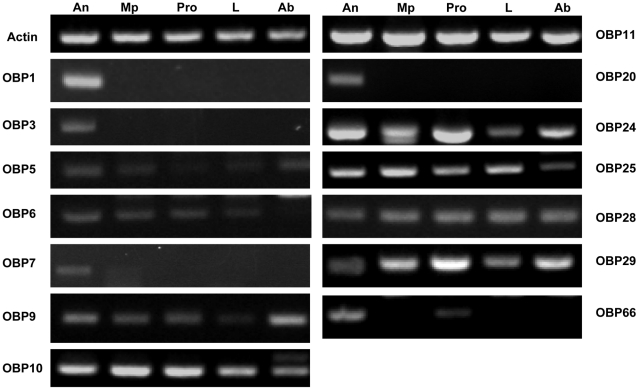
RT-PCR analysis of *An. funestus* OBPs in different tissues. An, antennae; Mp, maxillary palps; Pro, proboscis; L, Leg and Ab, abdomen. AfunOBP1, 3, 7 and 20 were detected only in the antennae while AfunOBP66 was detected in both antennae and proboscis.

Tissue specificity of fourteen *An. funestus* OBPs has been examined by RT-PCR in different tissues (olfactory tissues: antennae, maxillary palps, proboscis; non-olfactory tissues: legs and abdomens). Hitherto, most insect OBPs with a proven role in olfaction have been shown to be expressed exclusively in olfactory tissues. We hypothesize that an OBP gene abundantly and exclusively detected in chemosensory tissues likely encodes a “true” OBP [19]. In order to examine the transcripts levels between olfactory and non-factory tissues, amplification of a “house-keeping” gene encoding actin was used as control to check the integrity of each cDNA preparation. RT-PCR experiments showed that AfunOBP1, AfunOBP3, AfunOBP7 and AfunOBP20 were detected only from female antennae whereas AfunOBP66 was detected in both female antennae and proboscis (Fig. 6). On the other hand, remaining OBPs were detected in olfactory as well as non-olfactory tissues, indicating that they might not necessarily be involved specifically in olfaction.

We could not exclude that *AfunOBP* genes expressed in both olfactory tissues and non olfactory tissues also function in the olfactory system. However, it is likely that olfactory-specific *AfunOBP* genes are directly involved in olfactory mechanisms and represent “true” OBPs. For example, CquiOBP1, which was only detected in *Cx. quinquefasciatus* olfactory tissues [19], was shown to bind to a mosquito oviposition pheromone (MOP), 6-acetoxy-5-hexadecanolide [42], in a pH-dependent manner [43]. By immunohistochemistry experiment with a specific antibody, this protein was detected in a subset of trichoid sensilla including one type responding to this pheromone but neither in the grooved peg sensilla nor in the basiconica sensilla on the maxillary palps [43]. By using RNA interference (RNAi), reduction of CquiOBP1 transcription in female antennae led to significantly lower electrophysiological responses to MOP and other known mosquito oviposition attractants [23]. AgamOBP1, which has significant high mRNA concentrations in female vs. male heads and is down-regulated after a blood meal, was suggested to be involved in female *An. gambiae* host-seeking behaviors [44]. Indole was identified as the only ligand with affinity to AgamOBP1 by using *in silico* as well as biochemical assays [24]. RNAi gene silencing coupled with electrophysiological analyses further revealed that *An. gambiae* response to indole was abolished after the knock-down of AgamOBP1 [24]. Interestingly, putative orthologous genes of olfactory-specific AfunOBP1, 3, 7, 20 and 66 were also detected at very high levels and/or exclusively in the olfactory tissues of *An. gambiae* [15] and *Cx. quinquefasciatus* [19] indicating that these proteins are likely involved specifically in chemoreception across several mosquito species.

**Figure 7:**
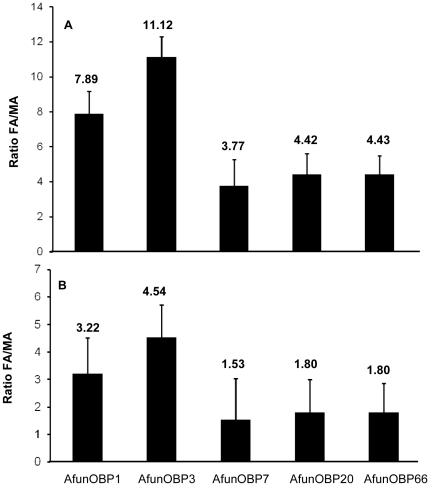
Quantitative real-time PCR (qPCR) analysis of *An. funestus* OBPs from female and male antennae. Normalized by (**A**) *actin* gene and (**B**) *AfunOR7*. Error bars show standard deviation. Significantly differentially expressed *AfunOBP* genes for female and male antennae distributions were determined as *p*-value <0.05 by student t-test.

Then, expression levels of olfactory-specific *AfunOBP1*, *3*, *7*, *20* and *66* were compared between female and male antennae by using quantitative real-time PCR (qPCR). Only female adult mosquitoes need a protein-rich blood meal to acquire nutrients necessary for eggs maturation after mating, while males do not feed on blood. Therefore, female specific or female enriched OBPs may be more specifically involved in host-seeking behavior. For such comparison, two different genes were used as endogenous control, *actin* and the OR83b-like odorant receptor cloned from *An. funestus* (*AfunOR7*). When actin was used as control, female antennae over male antennae expression ratios (FA/MA) of all OBP genes ranged from 3.77 to 11.12, indicating a general enrichment in female antennae relatively to male antennae (Fig. 7A). Two genes, *AfunOBP1* and *AfunOBP3* displayed the highest enrichment in female antennae with FA/MA ratios of 7.89 and 11.12, respectively. *AfunOBP7*, *20* and *66* ratios displayed comparable but lower enrichment in female antennae, around 4 times. When AfunOR7 was used as control, FA/MA ratios were significantly reduced for all genes tested (Fig. 7B). Again, *AfunOBP1* and *AfunOBP3* displayed the highest enrichment in female antennae with FA/MA ratios of 3.22 and 4.54, respectively, and other genes (*AfunOBP7, 20* and *66*) displayed comparable but lower enrichment in female antennae, around 1.5–1.8 times. Expression analysis by qRT-PCR demonstrates that two antennae-specific OBP genes, AfunOBP1 and AfunOBP3, are both enriched in female antennae. Similar study on gender ratios (female/male) was performed on *AgamOBP* genes by microarray and qPCR [44]. *AgamOBP1* and *AgamOBP3*, the orthologous genes of *AfunOBP1* and *AfunOBP3*, were also detected at higher levels in female antennae than in male antennae [44]. By using microarray, *AgamOBP3* showed a 9.2 times higher transcripts level in female antennae and AgamOBP1 showed a 4.2 times enrichment [44]. By using qPCR, *AgamOBP3* showed a 8.1 times higher transcripts level in female head than in male and *AgamOBP1* showed a 4.4 times enrichment [44]. Both approaches indicate that OBP1 and OBP3 are also female abundant OBPs in *An. gambiae* [44]. An *AfunOBP1* orthologous gene from the Asia malaria vector *Anopheles stephensi*, *AsteOBP1*, was also cloned and studied recently [45]. The level of AsteOBP1 transcript was 7-fold higher in female antennae than in male antennae by qRT-PCR, revealing that AsteOBP1 was also abundant in female *An. stephensi*.

### Sequence Analysis of AfunOBP1 and AfunOBP3

AfunOBP1 contains 144 amino acids. The 19 residues at the N-terminal were predicted as signal peptide (Table 1). The predicted mature AfunOBP1 showed 93% identity to AgamOBP1, 90% identity to CquiOBP1 and 85% identity to AaegOBP39. This OBP was initially isolated from antennae of the yellow fever mosquito, cloned, and named AaegOBP1 [46], but it was later renamed AaegOBP39 [18]. AfunOBP3 contains 151 amino acids. The 27 residues at the N-terminal were predicted as signal peptide (Table 1). The predicted mature AfunOBP3 showed 95% identity to AgamOBP3, 90% identity to AaegOBP38 and 87% identity to CquiOBP2. AfunOBP1 and AfunOBP3 shared 68% identity at amino acid level. Both of them belong to the OS-E/OS-F group (Fig. 5) and display the highest identity between orthologous proteins from different mosquito species (Table 2). Recombinant AfunOBP1 and AfunOBP3 were prepared by using a periplasmic expression system, which is known to generate properly folded, functional OBPs [47].

AgamOBP1, the orthologous OBP of AfunOBP1, undergoes a pH-dependent conformational change which is associated with a diminished capacity for binding [48]. The crystal structure of this protein suggests that the N- and C-termini of this protein may play a role in the reduction of binding by allowing these termini to unfold at low pH, thereby exposing the ligand to solvent [48]. The same phenomenon was also observed in the *Ae. aegypti* orthologous protein, AaegOBP1 ( = AaegOBP39 [46], [49]. Circular Dichroism (CD) and structural analysis indicated that AaegOBP1 ( = AaegOBP39) undergoes a pH-dependent conformational change, which may lead to release of odorant at low pH (as in the environment in the vicinity of odorant receptors). A C-terminal loop covers the binding cavity and this “lid” may be opened by disruption of an array of acid-labile hydrogen bonds thus explaining reduced or no binding affinity at low pH [49]. Based on their high identity at the amino acid level, it is very likely that AfunOBP1 also shares similar structure and pH-dependent conformational change mechanism with its orthologous protein in *An. gambiae* (AgamOBP1) and *Ae. aegypti* (AaegOBP1, later named AaegOBP39 [49]).

### Binding affinities of AfunOBP1 and AfunOBP3 towards odorant ligands

**Figure 8:**
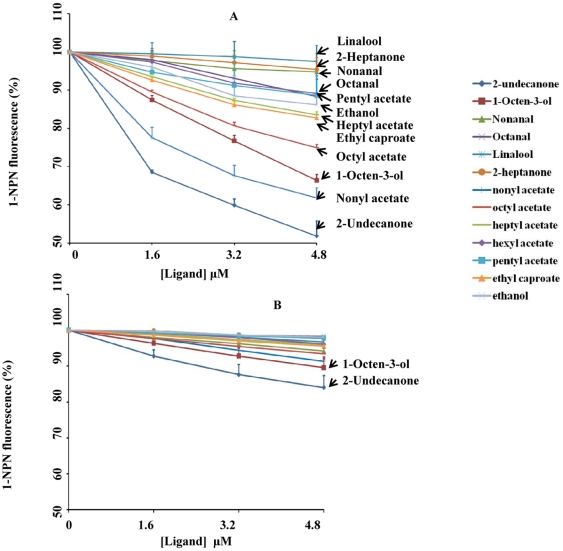
Binding curves of EAG active compounds to AfunOBP1 and AfunOBP3. Error bars show standard deviation. At pH 7, AfunOBP1 (A) significantly binds to 2-undecanone, nonyl acetate, octyl acetate and 1-octen-3-ol but AfunOBP3 (B) showed almost no binding activity to any of the selected EAG active compounds.

Here fluorescence binding assay was used to determine the binding activity of AfunOBP1 and AfunOBP3 to the EAG-active compounds. Insect OBPs are involved in the first step of odorant reception where they bind, solubilize and deliver odorant molecules to ORs [10]. We hypothesized that AfunOBP1 and AfunOBP3, two female most abundant olfactory tissue specific OBPs in this study, show binding affinity to some or all of these EAG active odorant compounds. The results showed that AfunOBP1 displays high selectivity towards different EAG-active ligands (Fig. 8A). The ligand with the highest affinity to AfunOBP1 was 2-undecanone, which is also the compound that elicited the highest EAG response per µmol of odorant. 2-Undecanone was first identified from the wild tomato [35] and later used as an insect repellent on mosquitoes such as *An. gambiae*, *Ae. aegypti* and ticks [36], [37], [38]. It is still unknown if 2-undecanone is also a repellent to *An. funestus*. AfunOBP1 also showed binding affinity to nonylacetate, octylacetate as well as 1-octen-3-ol. 1-Octen-3-ol was identified from both cattle [27] and human [25] and has been shown to serve as a powerful attractant for certain species of tsetse flies in the field [27]. Traps with 1-octen-3-ol have resulted in catches of only a few mosquito species, but in combination with CO_2_, an increase in the collections has been observed [31]. There were no reports on nonyl acetate or octyl acetate to mosquito behavioral study yet. Both are compounds identified from fruits and may be involved in mosquito sugar-feeding behaviors [34]. CquiOBP1, AfunOBP1 orthologous OBP in *Cx. quinquefasciatus*, was successfully used as molecular target based on its binding affinity to identify several *Culex* mosquito oviposition attractants in a reverse chemical ecology approach [43]. This study indicated that AfunOBP1 may be used in screening strategies for potential attractants as well as repellents for *An. funestus*, which may help in mosquito control.

**Figure 9:**
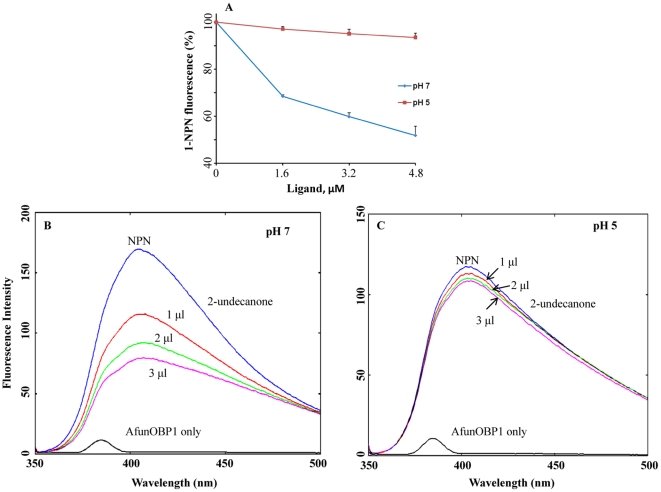
pH-dependent binding of 2-undecanone to AfunOBP1. Binding curves of 2-undecanone to AfunOBP1 at pH 7 and pH 5 (A) and binding affinity of 2-undecanone to AfunOBP1 as indicated by replacement of a fluorescence reporter, NPN at pH 7 (B) and pH 5 (C) showed that AfunOBP1 can bind 2-undecanone at pH 7 but not pH 5. Error bars show standard deviation.

On the other hand, AfunOBP3, which shares high identity (68%) with AfunOBP1, showed no binding affinity to any EAG active compounds (Fig. 8B). Two highly similar mosquito OBPs showed very different characteristics in their binding affinities (Fig. 8). X-Ray crystallography and nuclear magnetic resonance (NMR) have been successfully applied to solve the structure of insect OBPs from *Bombyx mori* (BmorPBP1) [50], [51], *D. melanogaster* (LUSH) [52], *Leucophaea maderae* (LmadPBP) [53], *Apis mellifera* (Amel-ASP1) [54], *A. polyphemus* (ApolPBP1) [55], *An. gambiae* (AgamOBP1) [48] *Ae. aegypti* (AaegOBP1) [49]. Comparative structural studies of AfunOBP1 and AfunOBP3 are certainly important in future research, particularly to address questions regarding molecular features of the pH-dependent conformational change observed for AfunOBP1. We have checked the effect of pH on the binding activity of AfunOBP1 to 2-undecanone (Fig. 9). AfunOBP1 showed high binding affinity to 2-undecanone at pH 7 but almost no binding affinity at pH 5. The same phenomenon was also observed on other insect OBPs. BmorPBP1 binds sex pheromone bombykol at pH 7 but not pH 5 [47], [56]. CquiOBP1 bound MOP at high pH but not low pH. Several studies have suggested that the membrane surface around the dendrite in insect sensilla is negatively charged [57], which induces a drop in pH in the close vicinity of receptors. A pH-dependent ligand release mechanism is likely to also apply in AfunOBP1.

### Conclusions

This work presents for the first time a study on the OBPs and odorants perceived by malaria mosquitoes *An. funestus*. By being highly anthropophilic and endophilic, *An. funestus* represents a very important malaria vector. Especially in recent years, it was suspected that due to the environmental changes or development of irrigation systems, favorable conditions have been created in Africa for the reestablishment of this dangerous species [58]. Here we performed a large EAG screening of over 50 ecologically significant odorant compounds and identified 12 ligands that elicit significant antennal response from *An*. *funestus* female antennae, and may function as attractants or repellents for *An. funestus*. We also applied a molecular approach based on known genome information of three mosquito species, *An. gambiae*, *Ae. aegypti* and *Cx. quinquefasciatus*, and successfully cloned 14 OBP genes from *An. funestus*. We demonstrated for the first time that two homologous OBPs, AfunOBP1 and AfunOBP3, sharing high identity at amino acid level and belonging to the same phylogenetic group (OS-E/OS-F), showed very different characteristics in their binding affinity. Binding assay experiments showed that AfunOBP1 significantly binds to 2-undecanone, nonyl acetate, octyl acetate and 1-octen-3-ol at pH 7 but AfunOBP3 showed nearly no binding activity to any of the selected odorant compounds at pH 7. These findings raise new questions: What changes on amino acid sequence cause these two AfunOBPs diverge in their characteristics in structures and binding affinities? What is the function of AfunOBP3? Addressing these questions will help us better understand the molecular mechanism and function of mosquito OBP genes. *An. gambiae* and *An. funestus* are both major malaria vector species in sub-Sahara Africa. If *An. comorensis* is excluded [59], *An. gambiae* s.s. is one member of a complex of 7 sibling species and *An. funestus* s.s. is one member of a group of 12 morphologically similar species some of which can be molecularly distinguished [60]. Members of the *An. gambiae* species complex and *An. funestus* species group, albeit being very closely related, have very different “biologies” that make some malaria vectors and others not. The interesting phenomenon to investigate is the genomic and biochemical parallels and differences that have evolved and allowed these two species to elicit such similar associations with humans. Both human blood feeding (anthropophily) and resting in human dwelling (endophily) behaviors are largely mediated by responses to chemicals in the environment. Comparative study on the olfactory systems of these two species will shed light on understanding the molecular mechanism that lead them to close association with humans and, therefore, could be of greatest interest in malaria control.

## Materials and Methods

### Mosquito rearing

*An. funestus* specimens used in this study were obtained from a sub colony established from the original FUMOZ colony [61]. Mosquitoes were maintained at 26°C, 85% RH with cycles of 12 hours light 10 hours dark with 1 hour dim light crepuscular periods in between to promote mating.

### EAG recording

One to six days old non-blooded *An. funestus* females, fed on 10% sucrose *ad-libitum*, were used. A head was excised with a sharp scalpel and placed on the reference electrode coated with electrode gel (Parker Laboratories, Orange, NJ). Distal end of the antennae (less than half a millimeter), cut to ensure a good electrical contact, were carefully placed on the recording electrode purchased from Syntech, Germany. EAG signals were fed to a 10× amplifier and processed with PC-based interface and software package (Syntech, Germany).

Preliminary experiment revealed that 1-octen-3-ol, a host derived kairomone identified from cows [27] and human sweat [25], elicits significant EAG response from female *An. funestus* antennae, as was also reported for *An. gambiae* [25], [26]. Thus 1-octen-3-ol was used as a reference compound in screening of a wide array of chemicals that included alcohols: hexanol, heptanol, nonanol, 1-hepten-3-ol, 4-methylcyclohexanol, 3-octanol, (*E*)-2-hexen-1-ol, linalool and 1-dodecanol; aldehydes: hexanal, heptanal, octanal, nonanal, decanal and undecanal; carboxylic acids: formic acid, acetic acid, propanoic acid, butanoic acid, hexanoic acid, heptanoic acid, octanoic acid, nonanoic acid, decanoic acid, isovaleric acid, lactic acid, trans-2-hexenoic acid, 5-hexenoic acid, (*E*)-3-hexenoic acid, oxobutyric acid, 2-oxovaleric acid, 7-octenoic acid and 3-methyl-2-hexenoic acid; esters: methyl acetate, ethyl acetate, propyl acetate, butyl acetate, pentyl acetate, hexyl acetate, heptyl acetate, octyl acetate, nonyl acetate, ethyl propionate, ethyl-3-hydroxyhexanoate, ethyl hexanoate; ketones: 2-butanone, 2,3-butanedione, 5-methyl-2-hexanone, 2-heptanone, 2-undecanone and 2-tridecanone; and phenols: 2- and 4-methyl phenol. Most of the chemicals were purchased from Sigma–Aldrich and were all ≥95% pure. 7-Octenoic acid and 3-methyl-2-hexenoic acid were kindly provided by Bedoukian Research Inc (Danbury, Connecticut). Formic acid (88%) and acetic acid (100%) were purchased from Fisher, USA. Formic acid, ethanoic acid, propanoic acid and butanoic acid were diluted in double distilled water, whereas all other compounds were diluted in distilled hexane. EAG responses were normalized by using 1-octen-3-ol as a reference (100%) and hexane was used as a control.

Pure chemicals were diluted 10 times to have a stock solution of 100 µg/µl from which decadic dilutions were made. A 10 µl aliquot of each solution was applied to a filter paper strip (1×3.5 cm; Whatman No. 1, Fisher Scientific) and the solvent was evaporated under a fume hood before inserting the paper strip into 5 ml disposable plastic syringe (Becton Dickinson, Franklin Lakes, NJ). A 500 ms pulse (5 ml/s) was delivered by stimulus controller CS-55 (Syntech, Germany) to deliver chemical stimulants to a humidified continuous air flow (10 ml/s) over the EAG preparation. The chemicals were tested randomly and applied with 0.5–1 min intervals between stimulations.

Initial screening was performed using a 100 µg source dose solution and chemicals that elicited up to 50% EAG response compared to 1-octen-3-ol were selected for further dose-response study. Three to twenty mosquitoes were tested for each compound to calculate the average EAG amplitude and standard deviation (STD) [25].

Volatility measurements were carried out on a gas chromatograph (GC) 6890 (Agilent Technologies) equipped with a capillary column (HP-5MS, 25 m×0.25 mm; 0.25 µm); helium was the carrier gas. Samples were injected in splitless mode; injector port and flame ionization detector (FID) temperatures were set at 250°C. The GC program was: 50°C for 1 min, increased to 150°C at a rate of 10°C/min and held at this temperature for 5 min. EAG syringes with stimuli aliquots on the filter paper were prepared in the same way as used in EAG analysis. After waiting for 10 min, a sample of 2 µl headspace was collected by a 10 µl gas-tight syringe (Agilent) and injected into GC for quantification. Each preparation was analyzed 10 times to obtain mean and standard deviation [41]. The amount of each studied chemical was calculated with a calibration curve, which was obtained by using standard solutions of the same compound at various concentrations.

### Molecular cloning of AfunOBPs

The genome of *An. funestus* is yet to be sequenced, whereas genome sequences of *An. gambiae*, *Ae. aegypti* and *Cx. quinquefasciatus* are already available. Therefore, homology cloning was used to identify AfunOBP genes. Degenerate and gene-specific primers were designed according to known mosquito classic OBP cDNA sequences (Table S1). Total RNA was prepared by using TRIzol (Invitrogen, Carlsbad, CA) from the late stage pupae mixture of male or female and cDNA was synthesized by using SMART RACE cDNA amplification kit (BD Sciences, Clontech, Palo Alto, CA) with SuperScript II reverse transcriptase (Invitrogen, Carlsbad, CA), according to the manufacturer's manual. PCR was performed in a GeneAmp PCR system 9700 thermal-cycler (Applied Biosystems, Foster City, CA) for 50 cycles with an annealing temperature of 55°C. PCR products were gel-purified using QIAquick gel extraction reagents (Qiagen, Valencia, CA), cloned into the pBluescript SK (+) cloning vector (Stratagene, Carlsbad, CA) and subsequently sequenced (http://www.davissequencing.com/sample_prep.htm). 3′ RACE (Rapid Amplification of cDNA End) PCR was performed according to SMART RACE cDNA amplification kit manual with universal primer and gene-specific primers (Table S2). 5′ RACE PCR was carried out by using SMART RACE cDNA amplification kit and 5′-Full RACE Core Set (Takara, Madison, WI) with specific primers (Table S2 and S3). PCR products were further cloned into the pBluescript SK (+) cloning vector and sequenced. The complete AfunOBPs nucleotide sequences have been deposited into Genbank under accession numbers given in Table 1.

### Bioinformatics analysis of mosquito OBPs

N-terminal signal peptides of AfunOBPs were predicted by using SignalP 3.0 (http://www.cbs.dtu.dk/services/SignalP). The calculated molecular weights and isoelectric points (pI) were obtained by using ExPASy proteomics server (http://www.expasy.org/tools/protparam.html). Blast in NCBI conserved domains database (CDD) was used to identify PBP/GOBP motifs. The amino acid sequence alignment of 14 AfunOBPs was performed by using clustal W2 (http://www.ebi.ac.uk/Tools/clustalw2/index.html). In this study, 14 new cloned AfunOBPs, 33 classic AgamOBPs [15], [16], [17], 34 classic AaegOBPs [18] and 53 classic CquiOBPs [19] amino acid sequences were used to create entry file for phylogenetic analysis in MEGA 4.0.2 [19]. An un-rooted consensus neighbor joining tree [62] was calculated at default settings with pair-wise gaps deletions as previously described [19].

### Expression profiles of An. funestus OBPs

Total RNA was isolated from the 4–6 days old adult female antennae, maxillary palps, proboscis, legs and abdomens by using TRIzol and cDNA was synthesized from 1 µg of RNA as described above. All subsequent PCR reactions were carried out using 40 cycles with gene specific primers (Table S4). All RT–PCR reactions were replicated at least three times. Furthermore, the *An. funestus* actin gene was amplified as a control for cDNA integrity by using the primers Actin-f and Actin-r (Table S4).

ABI 7300 Real Time PCR Instrument (Applied Biosystems, Foster City, California) was used with the Express SYBR@Green qPCR SuperMix Universal (Invitrogen) for quantitative real-time PCR. In each reaction, 10 µl of Supermix, 0.4 µl ROX Reference Dye (25 µM), 0.2 µM forward and reverse primers (Table S5), was added to individual wells of a 96-well plate to which 1 µl of cDNA was added as a template. The cycling parameters were: 50°C for 2 min, 95°C for 2 min; 40 cycles of 95°C for 15s, 60°C for 1 min. For each cDNA sample and primer set, reactions were run in triplicate and average fluorescence Ct values were obtained. Relative gene expression ratios between female and male mosquitoes were determined using the Pfaffl method of analysis [63].

### Protein expression, purification and circular dichroism analysis

pET22b vector was used for expression of recombinant AfunOBP1 and AfunOBP3 as described before [56]. Expression was performed in LB medium with transformed BL21 (DE3) cells (Novagen, San Diego, CA). Proteins in the periplasmic fraction were extracted with 10 mM Tris·HCl (pH 8) by using three cycles of freeze-and-thaw and centrifuging at 16,000×*g* to remove debris. The supernatant was loaded on a Hiprep™ DEAE 16/10 column (GE Healthcare). Unless otherwise mentioned, all separations by ion-exchange chromatography were done with a linear gradient of 0–500 mM NaCl in 10 mM Tris·HCl (pH 8). Fractions containing the target protein were further purified on a 20 ml Q-Sepharose Hiprep™ 16/10 column (GE Healthcare) and, subsequently, on a Mono-Q HR 10/10 column (GE Healthcare). OBP fractions were concentrated by using Centriprep-10 (Millipore) and loaded on a Superdex-75 26/60 gel-filtration column (GE Healthcare) pre-equilibrated with 150 mM NaCl and 20 mM Tris.HCl (pH 8). Highly purified protein fractions were concentrated by Centricon-10, desalted on four 5-ml HiTrap desalting columns (GE Healthcare) in tandem and by using water as mobile phase, analyzed by LC-ESI/MS, lyophilized, and stored at −80°C until use. The concentrations of the recombinant proteins were measured by UV at 280 nm in 20 mM sodium phosphate (pH 6.5) and 6 M guanidine HCl by using the theoretical extinction coefficient calculated with EXPASY software (http://us.expasy.org/tools/protparam.html).

### Fluorescence binding assay

N-phenyl-1-naphthylamine (1-NPN, also NPN) was used as a reporter ligand in fluorescence binding assay experiments. First the affinities of the 1-NPN to AfunOBP1 and AfunOBP3 were measured using 10 µg/ml protein solutions prepared in 20 mM ammonium acetate, pH 7. Increasing doses of 1-NPN (3.2 mM in ethanol, 0.5–2.5 µl) were added to the protein solutions until the fluorescence intensity reach a plateau. The amount of 1-NPN added was recorded and the fluorescence intensity was used as a reference (100%) to normalize the following measurements. For AfunOBP1 (10 µg/ml) at pH 7, 6.4 µM final concentration of 1-NPN was added to reach fluorescence intensity saturation while 3.2 µM final concentration of 1-NPN was added for AfunOBP3 (10 µg/ml) (Fig S1). Then one of the selected EAG-active ligands was added (3.2 mM, 1–3 µl; final concentrations, 1.6–4.8 µM) and the fluorescence intensities were recorded and normalized by using the NPN reference. Fluorescence measurements were done on a spectrofluorophotometer (RF-5301, Shimadzu, Kyoto, Japan) at 25±1°C. Samples in 2-ml cell were excited at 337 nm, and the emission spectra were recorded from 350 to 500 nm, with emission and excitation slit widths of 1.5 and 10 nm, respectively [43].

## Supporting Information

Table S1 **List of primers designed for screening** ***AfunOBP*** **genes.**
(PDF)

Table S2 **List of primers designed for cloning 3′ and 5′ RACE sequences of AfunOBP cDNA sequences.** SMART RACE cDNA amplification kit was used.
(PDF)

Table S3 **List of primers designed cloning 5′RACE sequences of AfunOBP cDNA sequences.** Takara 5′-Full RACE Core Set kit was used.
(PDF)

Table S4 **List of primers designed for** ***AfunOBP*** **genes RT-PCR analysis.**
(PDF)

Table S5 **List of primers designed for** ***AfunOBP*** **genes quantitative real-time PCR (qPCR) analysis.**
(PDF)

Figure S1 **Binding curves of 1-NPN to AfunOBP1 and AfunOBP3.** To 10 µg/ml AfunOBP1 (**A**) at pH 7, 3.2 µM of 1-NPN was needed to saturate the fluorescence intensity while fluorescence from 10 µg/ml of AfunOBP3 (**B**) was saturated with 1.6 µM of 1-NPN.
(TIF)
