# Supplementary material for: Odorant-Binding Proteins of the Malaria Mosquito Anopheles funestus sensu stricto
Source: PLoS One. 2010 Oct 22;5(10):e15403. doi: 10.1371/journal.pone.0015403 (PMC2962654; doi:10.1371/journal.pone.0015403)
Supplement: Table S1 — List of primers designed for screening AfunOBP genes. (PDF) [file pone.0015403.s001.pdf]

AgamOBPs	Forward Primer	Reverse Primer
1, 17	GCTSAARTGCTACATGRACTGTMTSTTCC	TTCCAGCACTTGTKMARCCAGAACGC
2	GCTCAAGTGCTACATGGA	CKKYTTCCAGCAYTTGTG
3	CTGAARTGYTACATGAACTG	TTCCAGCACTTRTTSAGCCAGAA
4	GCRATGACSATGAAACAGCT	CCTGCCATTTGTGCAATACACAT
5	TGAMCAAAAARGGCGAAAT	TYTTRTCGCAMGWKTCCTTGTA
6, 18	CTAACMCAAACWTCGAAAAAAGG	TTGGGSSTYGCTTTGAAGAA
7, 65	GCCGAGTCGGGCGCCTCGGAGGA	CCTTCACGTCGTCCGGGATGATGTA
8	ATGCCAAGCCGGAAACGATTG	GCGAGCTGCCCCGCCCGGCGTAC
9	GCGTACCGGGMCGAGTGCGT	GTCSARCAGSTSCRYCTTSTKSARGATGC
10	AGTGCTWYGTSCAATGYTTCTTC	CTTVGCYTTGTCYGCKTCCATCAG
11	CTGCTTCAACAMGCTGCGMATCCC	GGAAGCASAGCTCCTGCTTGTACGC
12	GCATTCTATCCCCTGTTTGGTTGTGCTCG	CAGTTGTTCAGCGTACGGCCTCGCAGTAC
13	GGCAATGGTCTGTCGGGTGCAGGCGGG	CTTACAGTCGGCCGCAATCTCCTTCGCC
14	AGAAAGCGTCCACCATCTTCG	CGGCCAGACACTGCTCGGCCTTCT
15,16	GCAAACGCTCCAAAGTCTCTGTCCCCGG	CGAGTTGAACTGCTTCGGAATGGCGTGG
19, 20	CARATYGACACRATSMTKCCGGAC	CTAYGGRAAAAYRAATTTWGGATTGTT
21	TGCSRKGCCGAGSTGGGCG	TGWRGTAGCACTSGTACAG
22	GGTAATGTTTAACGCTGTGCAGGGCGC	CGCACAGATCTTCGATTGTTTCCAG
23	GTGTATGGCCGAGACAGGCATTGGGGC	GATAGTGATCGTGATAGCACGCGTACGG
24, 25	AAGTGCTTKGTGAAATGYTTC	GTAKGCCGTSTCGCAMGCGTCCG
26,	GAYAAGACCAAGTGCTTC	CCTTSTGGTTGCACTTCTTCA
27	CCGTCAGGAACTCTCTCTGCTTTGGCG	CGTCGAACGCAGTGACGCAGTACGTCGG
28	GACCAGAWRAAGAAGGCSGAAGG	TGCACTTCTTCACCAGYSCCTCCACCTT
29	GTGCGATAGTGCTTCTGCTGCTGGTGGGC	GTGCTTCTCGGGAAACCCGTCCGCG
66	ACCGGMAGCTTCCCGGA	TCCTSCARACAVTCGTC
67	CTGGCSGGMAARTGYATGCA	CCCTGGAAGAAGCACTGCACGAAGCA
68	GAGTGCTTYACCGAGTGYCTGATGAA	AAYCCSGTYACGCAYACCGT

Table S1 List of primers designed for screening *AfunOBP* genes.
