# Supplementary material for: Odorant-Binding Proteins of the Malaria Mosquito Anopheles funestus sensu stricto
Source: PLoS One. 2010 Oct 22;5(10):e15403. doi: 10.1371/journal.pone.0015403 (PMC2962654; doi:10.1371/journal.pone.0015403)
Supplement: Table S2 — List of primers designed for cloning 3′ and 5′ RACE sequences of AfunOBP cDNA sequences. SMART RACE cDNA amplification kit was used. (PDF) [file pone.0015403.s002.pdf]

AfunOBPs	3’RACE	5’RACE
1	GGTCGACGACAATGGTGACGTGCATCTGG	
3	CTGGAGATGCCATCAAGCGGTTTAGTG	
5	GCGTTCGGCATGTGCTCCCAAGTTC	CGCAGGTTATCCAACATTTCGGTGG
6	GCAGATTGACATCCTTCCGGAAGAG	CATCAGCTGCAGTACGACAAGAATCG
7	GGACGCATCTTGTTGGATCGATTGC	GTTCCGTCCAAACATTGGCGCAGCAG
9	GGAGCAGTACAAATCGTGGAACTTCCCGG	
10	GAAGGACAAGCTGGAGGTGTTTCTCACC	ACGACACCCGTCTCATCCATCAGG
11	CCCATCCTACATGTCCGGTATCTTCCCGG	
20	GATGCGGGCGGCGCTCGACATCTGCCG	
24	CGGTGTGATTCAGCAGGATGTGATTCGGG	
25	GGTCAGCTGCAGGAGAAGACGATCACGG	
28	CAGGAACATCTGGTACTGCTCCGGGAC	
29	GATAAGTGTGATTACTCGATGCG	
66	TCGGCATCCTGACGGAGGACGACAAGG	

Table S2 List of primers designed for cloning 3’ and 5’ RACE sequences of AfunOBP cDNA sequences. SMART RACE cDNA amplification kit was used.
