# Supplementary material for: Odorant-Binding Proteins of the Malaria Mosquito Anopheles funestus sensu stricto
Source: PLoS One. 2010 Oct 22;5(10):e15403. doi: 10.1371/journal.pone.0015403 (PMC2962654; doi:10.1371/journal.pone.0015403)
Supplement: Table S3 — List of primers designed cloning 5′RACE sequences of AfunOBP cDNA sequences. Takara 5′-Full RACE Core Set kit was used. (PDF) [file pone.0015403.s003.pdf]

AfunOBP1 RT	CTTAGGATCAGATTGC	AfunOBP24 RT	GAACAGTTCCTTTGG
AfunOBP1-1	CGAGTTTGGCAGCGCATCGTGC	AfunOBP24-1	GAATCACATCCTGCTGAATCAC
AfunOBP1-2	GTCACCATTGTCGTCGACCACC	AfunOBP24-2	CGTCCTCGTCGATGAAACCGG
AfunOBP1-1R	GCAAACGTTGTCTCTATCCCGAG	AfunOBP24-1R	GATCTGATCAAGAAGTGTACC
AfunOBP1-2R	CTCTGCGACAAAGCCTTCTGGC	AfunOBP24-2R	GACGAGTGTGACACTGCCTATC
AfunOBP3RT	CACGTAATGAAACTC	AfunOBP25 RT	GGAAGTAACACTCCG
AfunOBP3-1	CTCTTCCATCAGGCGGGTGTGG	AfunOBP25-1	GAAGCAAGCGAGTCCAGCTTGAG
AfunOBP3-2	GTGTGGTGAACGATAAGGGTG	AfunOBP25-2	GGACGAGTGGATCCAAGCAC
AfunOBP3-1R	ACAGTTCATGTAACACTTCAG	AfunOBP25-1R	GCAGGAGAAGACGATCACG
AfunOBP3-2R	GAATGTCCTGATCACTAAACCGC	AfunOBP25-2R	GATGCGTGTGAAACCGCC
AfunOBP9RT	CCTTCTTCACGCTCG	AfunOBP28RT	GGACGCATCCATGAAG
AfunOBP9-1	GCACGATACTTCAGCAGATC	AfunOBP28-1	GTGGACGGTGAGACGAAGTGTTTC
AfunOBP9-2	GCACCACGAACTCAGCGCTC	AfunOBP28-2	CTGCGCTGCTTCCTGCACCAGGC
AfunOBP9-1R	CGATGTGGAT GAGGTGCGTG	AfunOBP28-1R	CCTGGGTCAGCCCTATATGC
AfunOBP9-2R	CCAACACCGACGGTAACGTG	AfunOBP28-2R	CCGAGCGCAAACCCTTCCGCCTTC
AfunOBP11RT	GCTGCACTTGGCCTG	AfunOBP29RT	CGCATCGAGTAATCAC
AfunOBP11-1	CAAACTGTACGCAGTGTCGATCG	AfunOBP29-1	CATCGACTCTATACAGGAGTG
AfunOBP11-2	CAGTACACGCCGAGCTTGATGG	AfunOBP29-2	CTGGCGCTGGACAATCGGCTG
AfunOBP11-1R	GCAGCAGAACTCACTACCGTGC	AfunOBP29-1R	CATCCGCGTTGGTGGCTTTCAG
AfunOBP11-2R	CAAGCAGGAGCTGTGCTTCCAG	AfunOBP29-2R	CGAAGAAACACTGCATCAGACAG
AfunOBP20RT	CCAGCACAATACTAGG	AfunOBP66 RT	GTGCCAGCGCCGTC
AfunOBP20-1	CAGTTGTTCTTGATACCGTC	AfunOBP66-1	CCGTTGTGCTTCATCCGGTGC
AfunOBP20-2	CACATTTCGGCAGATGTCG	AfunOBP66-2	CCTGACGGAGGACGACAAGG
AfunOBP20-1R	GATGCAGCATACGCCATGCTTC	AfunOBP66-1R	GTACGGTTGCACTGCATCAC
AfunOBP20-2R	CCATGCTTCAGTGCCTTTG	AfunOBP66-2R	CGTCCTGCATGGTGATTTCGG

Table S3 List of primers designed cloning 5’RACE sequences of AfunOBP cDNA sequences. Takara 5'-Full RACE Core Set kit was used.
