# Supplementary material for: Odorant-Binding Proteins of the Malaria Mosquito Anopheles funestus sensu stricto
Source: PLoS One. 2010 Oct 22;5(10):e15403. doi: 10.1371/journal.pone.0015403 (PMC2962654; doi:10.1371/journal.pone.0015403)
Supplement: Table S4 — List of primers designed for AfunOBP genes RT-PCR analysis. (PDF) [file pone.0015403.s004.pdf]

	Forward Primer	Reverse Primer
Actin	AAYTGGGAYGAYATGGARAA	GCCATYTCYTGYTCRAARTC
AfunOBP1	ATGAAGTTTGCTACATTCGTTTGTATCG	CAGATGCACGTCACCATTGTCGTCGACCACC
AfunOBP3	GCCATCAAGCGGTTTAGTGATCAGG	CTATGGTAAAAAGTAATGTAC
AfunOBP5	GCGATGACCCGCAAACAGTTGATC	GCTAGGGAAACAGAAACACTTAACG
AfunOBP6	CTTTGCTATCAACCTGCAGCAGTAGTGG	TAGGGGAAAAAGAACTTTGGGC
AfunOBP7	CCAACATACTGGTCGTCACCTTCACCG	TCACATTCATACCATCACCAGAAGGTG
AfunOBP9	GGAGCAGTACAAATCGTGGAACTTCCCGG	GCAAAGATCACTTTATTCCGTTCCG
AfunOBP10	CAAAAGGAGCTGACCGCACTGCC	GGGAATGTTTTCGCTTCTTTTA
AfunOBP11	CCCATCCTACATGTCCGGTATCTTCCCG	CCTGATGCATCAGTCCCACCAGCTC
AfunOBP20	GAACTGGCGGGACCGATGCGGGCG	GACACCAACATATGTACAGGACGCC
AfunOBP24	ATGAGGTGCAACGAATCGATCGTAACC	ACTTCTTGATCAGATCGTTCACCTTGCCC
AfunOBP25	CTTAGTGTTTGCTAGTGTGTTAAGTGTG	ATCAGTGATTCAATGCGCTC
AfunOBP28	GGCGGAAGGGTTCGCGCTCGGTTG	TCACAGCAGCGATGCCTTCTCCCGATG
AfunOBP29	CGGACGCCCTCCTCCACAGTTTGGC	CTAAAACATGGCAGACGTCACCG
AfunOBP66	GGATGCCCGAAATCACCATGCAGG	CTACTTATTATCCTTGCTCTTCCCATC

Table S4 List of primers designed for *AfunOBP* genes RT-PCR analysis.
