# Supplementary material for: Odorant-Binding Proteins of the Malaria Mosquito Anopheles funestus sensu stricto
Source: PLoS One. 2010 Oct 22;5(10):e15403. doi: 10.1371/journal.pone.0015403 (PMC2962654; doi:10.1371/journal.pone.0015403)
Supplement: Table S5 — List of primers designed for AfunOBP genes quantitative real-time PCR (qPCR) analysis. (PDF) [file pone.0015403.s005.pdf]

	Forward Primers	Reverse Primers
AfunOBP1	TGGGCAAACGTTGTCTCTAT	AGATTGCTTCCAGCACTTGT
AfunOBP3	ATCGATGCACCTTATTACGC	CGTTTTCCAACATTTGTTCA
AfunOBP7	CTCCGGATAAATGTGACACC	TCATTCCATCACCAGAAGGT
AfunOBP20	AACTGCGTGATGGAAATGAT	GCATGATTGTGTCGATCTGT
AfunOBP66	GACCGTATACCGTTGTGCTT	CCGTCTCCTTGTTAACCTTG
Actin	TAAAAATGATCTCGGCGAAC	GATCCACATCTGCTGGAATG
AfunOR7	CAGGTTTTCCTGTTTTGCAT	ACGAAGGTTTTTGCTTCCTC

Table S5 List of primers designed for *AfunOBP* gene quantitative real-time PCR (qPCR) analysis.
