# Supplementary figures and images for: Odorant-Binding Proteins of the Malaria Mosquito Anopheles funestus sensu stricto
Source: PLoS One. 2010 Oct 22;5(10):e15403. doi: 10.1371/journal.pone.0015403 (PMC2962654; doi:10.1371/journal.pone.0015403)

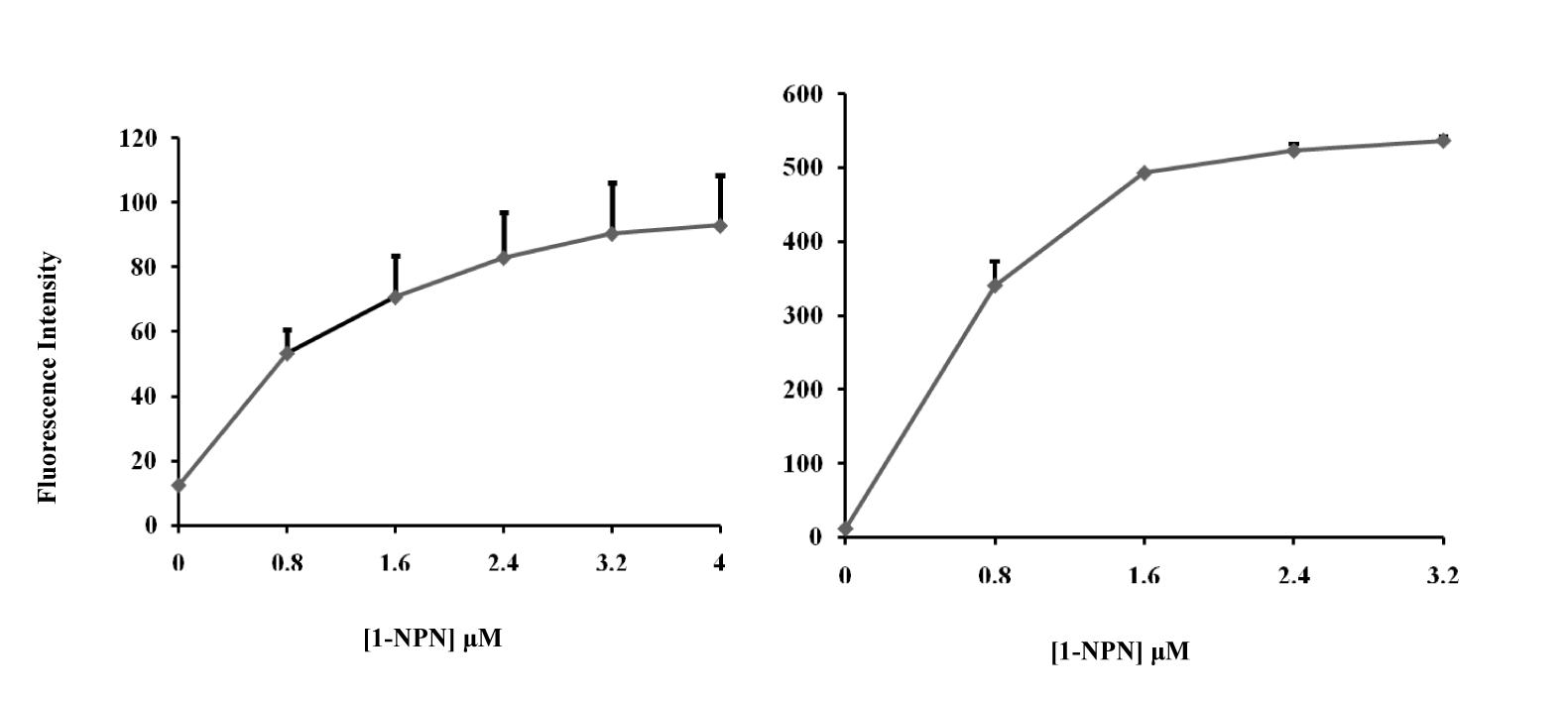

Supplement: Figure S1 — Binding curves of 1-NPN to AfunOBP1 and AfunOBP3. To 10 µg/ml AfunOBP1 (A) at pH 7, 3.2 µM of 1-NPN was needed to saturate the fluorescence intensity while fluorescence from 10 µg/ml of AfunOBP3 (B) was saturated with 1.6 µM of 1-NPN. (TIF) [file pone.0015403.s006.tif]
